# Biodegradation of Polycyclic Aromatic Hydrocarbons by *Novosphingobium pentaromativorans* US6-1

**DOI:** 10.1371/journal.pone.0101438

**Published:** 2014-07-09

**Authors:** Yihua Lyu, Wei Zheng, Tianling Zheng, Yun Tian

**Affiliations:** 1 Key Laboratory of the Ministry of Education for Coastal and Wetland Ecosystems, Xiamen University, Xiamen, China; 2 State Key Laboratory of Marine Environmental Science, Xiamen University, Xiamen, China; University of Kansas, United States of America

## Abstract

*Novosphingobium pentaromativorans* US6-1, a marine bacterium isolated from muddy sediments of Ulsan Bay, Republic of Korea, was previously shown to be capable of degrading multiple polycyclic aromatic hydrocarbons (PAHs). In order to gain insight into the characteristics of PAHs degradation, a proteome analysis of *N. pentaromativorans* US6-1 exposed to phenanthrene, pyrene, and benzo[a]pyrene was conducted. Several enzymes associated with PAHs degradation were identified, including 4-hydroxybenzoate 3-monooxygenase, salicylaldehyde dehydrogenase, and PAH ring-hydroxylating dioxygenase alpha subunit. Reverse transcription and real-time quantitative PCR was used to compare RHDα and 4-hydroxybenzoate 3-monooxygenase gene expression, and showed that the genes involved in the production of these two enzymes were upregulated to varying degrees after exposing the bacterium to PAHs. These results suggested that *N. pentaromativorans* US6-1 degraded PAHs via the metabolic route initiated by ring-hydroxylating dioxygenase, and further degradation occurred via the *o*-phthalate pathway or salicylate pathway. Both pathways subsequently entered the tricarboxylic acid (TCA) cycle, and were mineralized to CO_2_.

## Introduction

Polycyclic aromatic hydrocarbons (PAHs) are aromatic hydrocarbons with two or more fused benzene rings, and originate from natural as well as anthropogenic sources. They are widely distributed environmental contaminants that have detrimental biological effects, including toxicity, mutagenicity, and carcinogenicity [1], [2]. PAHs in the environment are degraded via volatilization, photo-oxidation, chemical oxidation, bioaccumulation, and adsorption to soil particles; however, the principal removal processes are thought to be microbial transformation and degradation [3]–[4]. In several microorganisms, metabolic pathways have been identified that lead to complete mineralization of aromatic hydrocarbons or partial transformation to dead end intermediates. As bioremediation efforts aggressively attempt to employ microorganisms to biostimulate or bioaugment degradation of aromatic hydrocarbons on a practical scale [5], the enzymes involved in the degradation of PAHs should be fully characterized as part of a risk assessment of this process.

PAHs are thermodynamically stable and resistant to microbial degradation due to their aromatic nature, which includes stabilization by multiple rings and low aqueous solubility. Despite these properties, a variety of bacterial species can degrade PAHs. Most research on the enzymes involved in PAH metabolism and genetic regulation is focused on *Pseudomonas* and *Sphingomonas* species [6]–[11], but *Mycobacterium*, *Rhodococcus*, *Nocardioides*, and *Novosphingobium* species can also mineralize PAHs [12], [13]. Proposed pathways for pyrene biodegradation by mycobacteria have been reported [14], including the complete pathway to the tricarboxylic acid (TCA) cycle proposed for *Mycobacterium vanbaalenii* PYR-1, but little is known about the genetic and enzymatic processes involved in the PAHs degradative pathways used by *Novosphingobium* species.

*Novosphingobium pentaromativorans* US6-1, is a marine bacterium that was isolated from muddy sediments of Ulsan Bay, Republic of Korea. Prior research has shown that it can degrade or transform phenanthrene, pyrene, chrysene, benz[a]anthracene, benzo[b]fluoranthene and benzo[a]pyrene. Because of its metabolic versatility, this bacterium was proposed to be a potential candidate for the bioremediation of PAH-contaminated areas [15]. In the current study, a proteomic analysis of cells exposed to phenanthrene, pyrene, and benzo[a]pyrene was conducted using one-dimensional gel electrophoresis in combination with liquid chromatography-tandem mass spectrometry to identify the proteins involved in the degradation. These analyses documented protein composition profiles in response to exposure to three different PAHs, and identified specific proteins that characterize the physiological state of *Novosphingobium*. *pentaromativorans* US6-1. A culture-independent method using reverse transcription and real-time quantitative PCR showed that gene expression of these different proteins was important in the biodegradation of PAHs. The results presented here comprise the foundation of a protein index for *N. pentaromativorans* US6-1, and provide fundamental information on PAH degradation as well as other metabolic characteristics in environmental strains of *Novosphingobium* sp.

## Materials and Methods

### 1 Bacterial strain and culture conditions

*Novosphingobium pentaromativorans* US6-1 used in this study was provided by Dr. Kim Sangjin (Korean Ocean Research and Development Institute). All chemicals used were purchased from Sigma-Aldrich unless otherwise specified, and were of high-performance liquid chromatography grade. The PAH compounds (phenanthrene, pyrene, and benzo[a]pyrene) were of >97.0% purity, and stock solutions of each PAH were prepared in cyclohexane at a concentration of 5 mg mL^−1^. *Novosphingobium pentaromativorans* US6-1 was cultured in Zobell 2216E broth (5 g L^−1^ peptone, 1 g L^−1^ yeast extract, 0.01 g L^−1^ FePO_4_, at pH 7.6–7.8) for 36 h at 25°C until exponential growth was achieved. Cells were collected via centrifugation and washed twice with MM2 medium [18 mM (NH_4_)_2_SO_4_, 1 µM FeSO_4_·7H_2_O, 100 µL 1 M KH_2_PO_4_/Na_2_HPO_4_ buffer solution and 10% MCD (mixed-cyclodextrin), per liter of aged sea water, at pH 7.2]. The cyclohexane was allowed to volatilize by storing it in the fume hood for 2–3 days at room temperature, then the bacterial inocula were added to 100 mL MM2 with 10 ppm phenanthrene, pyrene, and benzo[a]pyrene, at a final optical density of approximately OD value 0.3 (measured spectrophotometrically at 600 nm). The cultures were incubated at 30°C in the dark, and mixed using a rotary shaker at 150 rpm. The media containing the same amount of PAHs but without the bacterial inoculation was used as the control to determine any abiotic loss of PAHs. Triplicate samples were collected from the experimental system every 12 h for two days.

### 2 Analysis of PAH biodegradation

The residual PAHs were extracted using the liquid-liquid extraction method, followed by GC-MS determination. Fluoranthene was used as an internal standard to adjust the percent extracted. These samples were treated with equivalent dichloromethane for 15 min in an ultrasonic bath, filtered through anhydrous sodium sulfate, and concentrated by rotary evaporation to 1 mL under a gentle stream of nitrogen. PAH analysis was performed with a CP3800 gas chromatograph with a split/splitless injection port. The GC was equipped with a CP5860 silica capillary column (30 m×0.25 mm i.d., 0.25 µm film thickness) and helium carrier gas. The injection temperature was 300°C with an injection volume of 1 µL (splitless). The temperature program was as follows: isothermal at 100°C for 1 min, with 20°C/min to 200°C, 10°C/min to 320°C, and a final isothermal cycle at 320°C for 3 min. Quantification of PAH concentration was assessed with a GC/HRMS using a Varian 1200 (Palo Alto, USA) in the EI positive ion setting, with an electron energy of 70 eV. Transfer line temperature and ion source temperature were maintained at 250°C and 200°C, respectively, and the mass spectra obtained were compared to the NIST library.

### 3 Measurement of electron transport system activity (ETSA)

The measurement procedure for bacterial ETSA followed established methods [16]: 0.5 mL of culture was combined with 1 mL of 2-p-iodophenyl-3-p-nitrophenyle-5-phenyl tetra-zolium chloride, and allowed to interact for 30 min at room temperature (25°C) in the dark, then 100 µL of formalin was added to stop the reaction. The mixture was centrifuged (10,000×*g* for 5 min) in a Hitachi SCR20BC centrifuge, the supernatant was removed, and 1 mL methanol was added to the reaction tube. After the mixture was homogenized, centrifugation for 5 min at 10,000×*g* with a micro-12 centrifuge was repeated, and the reaction tube was placed in an ice bath. Absorbance was measured at 495 nm using a spectrophotometer. The ETSA in µgO_2_/mL/min was calculated using the following formula:
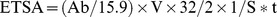
where Ab is absorbance; V is final reaction volume (mL) (methanol volume); S is the sample amount (mL); t is the incubation time (min); 32/2 is a constant (relative molecular weight of oxygen); and 15.9 is the molar absorbance of formazan.

### 4 Extraction of total proteins

After incubation for 24, 36, or 48 h, the medium dosed with phenanthrene, pyrene, or benzo[a]pyrene was carefully removed by centrifugation at 4,000×*g* for 5 min at room temperature. Cells were washed twice with 30 mL of PBS (pH 7.6) and pelleted by centrifugation under the same conditions. For protein extraction, the cell pellets were resuspended in 2 mL lysate (7 M urea, 2 M thiourea, 4% w/v 3-[3-cholamidopropyl dimethylammonio]-1-pro-panesulfonate [CHAPS], 60 mM dithiothreitol, 2% v/v Pharmalyte 3–10, and a protease inhibitor cocktail) and sonicated on ice for 10 min using a 500 ms/s pulse at 40 W. The sonicated cell suspensions were then centrifuged at 10,000×*g* for 30 min at 4°C. Protein concentration was estimated using the 2-D Quant Kit (Amersham, USA).

### 5 Electrophoresis and protein identification

SDS-PAGE was carried out using the tris-glycine-SDS buffer system (25 mM Tris, 192 mM glycine, and 0.1% SDS) on the SE600 electrophoresis apparatus (GE Healthcare, USA), at 24 mA/gel, until the dye front reached the bottom edge of the gel. A protein MW marker (Takara, China) was used in the gel, which was stained with Coomassie Brilliant Blue G-250 (GE Healthcare, USA), and scanned using an Amersham Biosciences image scanner (GE Healthcare, USA).

Three specific bands differently expressed under phenanthrene, pyrene, and benzo[a]pyrene were excised from the gel and used for in-gel tryptic digestion [17]. The gel pieces were destained and washed, and, after dithiothreitol reduction and iodoacetamide alkylation, the proteins were digested with trypsin for 20 h at 37°C. The resulting tryptic peptides were extracted from the gel pieces with 60% acetonitrile, 0.1% trifluoroacetic acid, and 100% acetonitrile. Extracts were dried completely for nano-liquid chromatography tandem mass spectrometry (LC-MS/MS) analysis. After resuspension in 20 µL 0.1% formic acid, the peptide samples were injected onto a Zorbax 300SB C18 peptide trap (Agilent Technologies, USA), and desalted with 0.2% formic acid at a flow rate of 10 µL min^−1^ for 20 min. Peptides were eluted from the trap and separated on a reverse phase C18 column (0.15 mm×150 mm, Column Technology), with a linear gradient of 4% to 50% mobile phase B (0.1% formic acid-84% acetonitrile) in mobile phase A (0.1% formic acid), over 30 min at 65 µL min^−1^. LC-MS/MS measurements were made with a linear trap quadrupole (LTQ) mass spectrometer (Thermo Finnigan, USA) equipped with a microspray source. The LTQ mass spectrometer operated in the data-dependent mode with the following parameters: spray voltage (3.4 kV), spray temperature 170°C, full scan m/z range (400–1800). The twenty most intense ions in every full scan were automatically selected for MS/MS.

### 6 Extraction of total RNA

At each of the aforementioned sampling times, total RNA was extracted from 100 mL of bacterial culture using Trizol (Invitrogen, USA), according to the manufacturer's instructions. Residual DNA was digested for 2 h at 37°C in a total volume of 200 µL using 100 U of DNase I in 40 mM Tris-HCl (pH 7.5) containing 6 mM MgCl_2_. DNase I was removed using standard phenolization and precipitation procedures [18]. The RNA pellet was dissolved in 200 µL RNase-free H_2_O and stored at −20°C. Extract concentrations were measured spectroscopically and were adjusted for optimal reverse transcription (RT)-PCR amplification.

### 7 RT-PCR and real-time quantitative PCR

For reverse transcription PCR (RT-PCR) of the isolated RNA, amplification reactions contained the following: 2 µg RNA template, 1 µL random hexamer primer (150 ng/µL), and 12 µL of nuclease-free water. Reactions were denatured for 5 min at 65°C, then 2 µL dNTP (10 mM), 4 µL 5×reaction buffer, 1 µL (20 U) RNase inhibitor, and 1 µL (25 U) RevertAid Reverse Transcriptase were added to obtain a total reaction volume of 20 µL. After centrifugation, the reaction was incubated for 5 min at 25°C followed by 60 min at 42°C, then terminated by heating the samples at 70°C for 5 min. RT-PCR analyses were completed using a Rotor-Gene 6000 (Corbett Research, Australia).

The sequences of the studied proteins, PAH ring-hydroxylating dioxygenase alpha subunit (RHDα) and 4-hydroxybenzoate 3-monooxygenase, were obtained from the GenBank nucleotide database (http://www.ncbi.nlm.nih.gov/nucleotide/) [19]. Primer Premier5 software (Premier Biosoft, Canada) was used to design a series of oligonucleotides from which three sets of specific primers (Table 1) were selected. The 16S ribosomal RNA (rRNA) gene was used as an internal standard. The relative quantification of genes by real-time quantification PCR was performed in a 12 µL reaction, which contained 2 µL of cDNA, 6 µL SYBR Premix ExTaq (TaKaRa, China), and 0.5 µL (10 µM) primer. Amplifications were carried out with the following temperature profiles: activation for 20 s at 95°C, followed by 40 cycles of 10 s at 95°C, 20 s at 60°C, and elongation at 72°C. Data was collected during each annealing step. Analyses of the dissociation curve and electrophoresis products were used to determine the specificity of the real-time PCR product. Each run included a negative control and a cDNA reaction without reverse transcriptase. Melt curve analysis was conducted at the end of each RT-PCR program, which measured signal intensity every 10 s as temperatures increased in 0.5°C increments from 51°C to 95°C.

**Table 1:** Characteristics of PCR primer sets used in this study.

Primer	Target gene	Sequence 5′-3′	Amplicon size (bp)	Reference
PAH-RHDα Fw	RHDα	GGA AAG GCT TGT GGG TGT CG	252	gi|494073952|
PAH-RHDα Re	RHDα	GTG GCA TCA TCG CAT CGT GT	252	gi|494073952|
HBMO Fw	4-hydroxybenzoate 3-monooxygenase	GCG TGT GCC GCC TTG TAA TCA	157	gi|494072487|
HBMO Re	4-hydroxybenzoate 3-monooxygenase	ACG CCA GTT CGT CCC AGA TGC	157	gi|494072487|
968R	16S rRNA	AAC GCG AAG AAC CTT AC	433	Felske A [20]
1401F	16S rRNA	CGG TGT GTA CAA GAC CC	433	Felske A [20]

The relative expression ratio of each target gene was calculated using the PAHs-treated group signal intensity divided by the control group signal intensity, after normalization to the levels of the standard gene. All analyses were based on the threshold cycle (C_t_), defined as the PCR cycle at which the fluorescence signal crossed a threshold line in the exponential phase of the amplification curve. Melting curve analysis of amplification products was performed at the end of each PCR reaction to ensure that only one product was amplified and detected. To maintain consistency, the baseline was set automatically by the software. A comparative C_t_ method was used to analyze the expression level of target genes, and the C_t_ for the target amplification of genes and the internal standard (16S rRNA gene) were determined for each sample. Differences between the C_t_ of the target and the internal standard, called ΔC_t_, were calculated to normalize the differences in the amount of total nucleic acid added to each reaction, and the efficiency of the RT-PCR. The control group (without PAHs exposure) was used as the reference sample, and was referred to as the calibrator. The ΔC_t_ for each sample was subtracted from the ΔC_t_ of the calibrator; this difference was called the ΔΔC_t_ value. The expression level of target genes could be calculated by 2-ΔΔC_t_, which represented the n-fold difference relative to the calibrator. In addition, template quantitation of test cDNA using the 16S rRNA gene was attempted. These analyses confirm the efficiency of the qPCR analysis.

## Results and Discussion

### 1 Biodegradation of PAHs by *Novosphingobium pentaromativorans* US6-1

**Figure 1:**
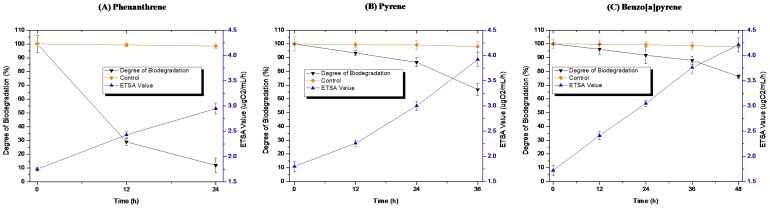
ETSA values and biodegradation rate of phenanthrene (A), pyrene (B), and benzo[a]pyrene (C) by *Novosphingobium pentaromativorans* US6-1 (mean and standard deviation of triplicate samples are shown.).

To gain a better understanding of the metabolic capabilities of *N. pentaromativorans* US6-1, the ETSA and biodegradation rates of phenanthrene, pyrene and benzo(a)pyrene were measured. The ETSA value provides an accurate measure of the actual respiratory rate of *N. pentaromativorans* US6-1 under different PAHs induction, and is positively positive correlated with cell activity. As shown in Fig. 1, the residual rate of PAHs decreased at different levels. After only 24 h of incubation, US6-1 degraded almost 86.62% of the phenanthrene. After 36 h of incubation, US6-1 degraded 31.81% of the pyrene, and after 48 h incubation degraded 22.18% of the benzo(a)pyrene. Along with the decrease in these residues, we observed an increase in ETSA to 2.95 µgO_2_/mL/min, 3.92 µgO_2_/mL/min, and 4.21 µgO_2_/mL/min at incubation times of 24 h, 36 h, and 48 h, respectively. However, in the control, which contained these three PAHs but no bacteria, phenanthrene, pyrene, and benzo(a)pyrene residues only decreased by 1.5%, 1.3%, and 1.4%, respectively.

HMW-PAHs, such as pyrene, benzo(a)anthracene, chrysene, and benzo(a)pyrene, are resistant to microbial attack. Most reported microbial degradation rates of pyrene and benzo(a)pyrene are quite modest in comparison to certain reported strains; e.g., *Mycobacterium* sp. strain PYR-1 degraded 90% phenanthrene after 14 days of incubation [21]. In previous reports, pyrene and benzo[a]pyrene were degraded by *Sphingomonas aromaticivorans* B0695 after incubation times of 28–32 h [22], *Burkholderia cepacia* degraded 20–30% of benzo(a)pyrene in the presence of a pyrene substrate after 63 days of incubation time [23], and consortium BL co-metabolized 44.07% of 10 ppm benzo(a)pyrene after an incubation time of 14 days [24]. PAHs biodegradation pathways in bacteria are induced by the presence of either the parent compound or one of the pathway intermediates. In our study, *N. pentaromativorans* US6-1 degraded phenanthrene, pyrene, and benzo(a)pyrene after 24 h, 36 h, and 48 h, respectively, suggesting that the degradation pathways were active in US6-1 during all treatments.

### 2 Comparison of the overall proteome profile in *N ovosphingobium pentaromativorans* US6-1

**Figure 2:**
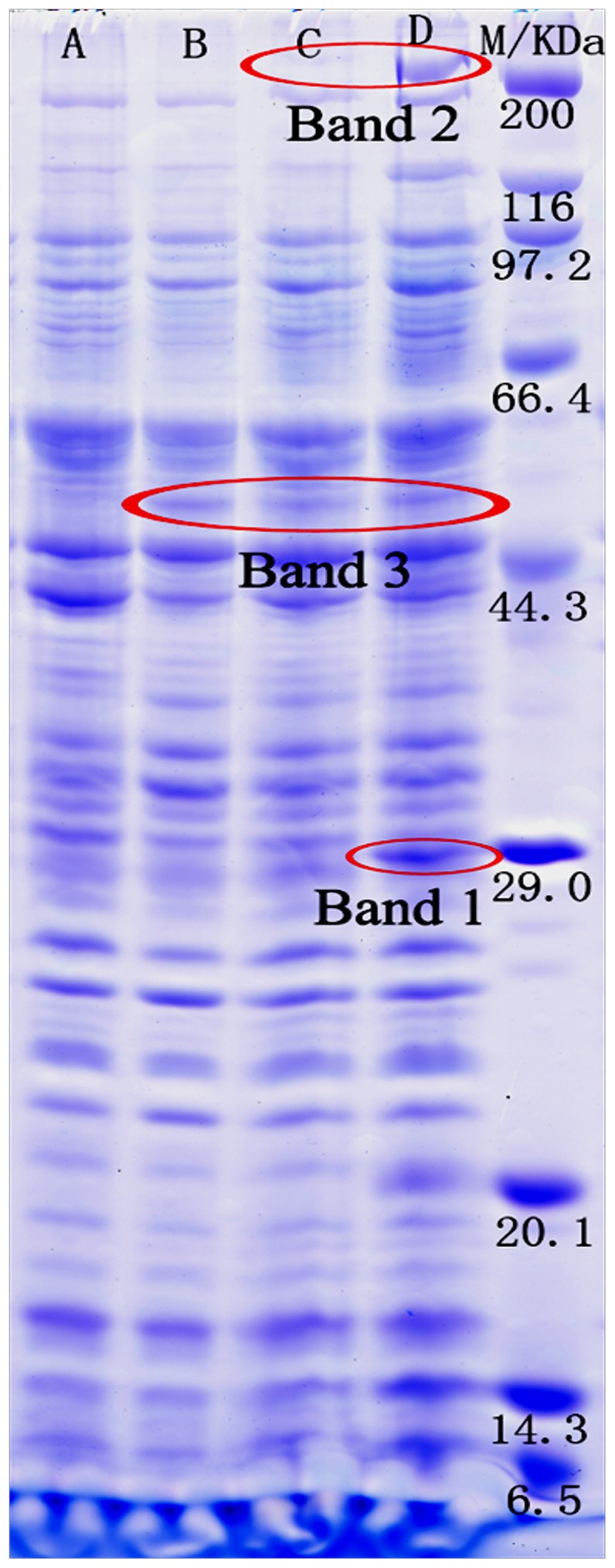
Separation of the total proteins of *Novosphingobium pentaromativorans* US6-1 using SDS-polyacrylamide gel electrophoresis. M, marker; A, control; B, phenanthrene-induced; C, pyrene-induced; D, benzo[a]pyrene-induced.

In order to characterize the molecular characteristics involved in the biodegradation of PAHs, the proteome profiles of *N. pentaromativorans* US6-1 induced by phenanthrene, pyrene, and benzo(a)pyrene were compared. The total protein profile of strain US6-1 (separated by SDS-PAGE) is shown in Fig. 2. Many enzymes involved in PAH degradation are likely to be expressed constitutively with so-called housekeeping proteins, and are not necessarily induced; therefore, the proteome profiles and band intensities were similar between the PAH-induced groups and the control. However, three specific bands were expressed differently after exposure to the three different PAH compounds. Compared with the control, Band 1 was upregulated only in the benzo[a]pyrene induced culture, Band 2 was intensified in both the pyrene and benzo[a]pyrene induced cultures, and Band 3 was expressed in the cultures treated with each of the three PAH compounds. As indicated by MW markers, the approximate MWs of Bands 1 and 2 were 29 KDa and 50 KDa, respectively, while that of Band 3 was about 200 KDa.

These three differential protein bands (Bands 1, 2, and 3) were excised and identified (Table 2). Unique proteins were found, but the peptide counts and percent cover were low; thus, it is possibly that although there were peptides in the bands, the integrated proteins were broken during the sample treatment. In Band 1, site-specific DNA-methyltransferase (adenine-specific), 3-oxoacyl-(acyl-carrier-protein) reductase, 4-hydroxybenzoate 3-monooxygenase, and homoserine kinase were identified. The site-specific DNA-methyltransferase (adenine-specific) is a type of transferase enzyme that transfers a methyl group from an S-adenosyl methionine to adenine to form N6-methyladenine. DNA methylation plays a key role in DNA stability, and is linked to the regulation of cell growth and gene expression [25]. Benzo(a)pyrene is a moderately potent carcinogen, and its reactive intermediates can covalently bind to cellular macromolecules (i.e., DNA) leading to mutations, thus, it is reasonable to hypothesize that this DNA-methyltransferase may be related to self-protection against mutation of the microorganism.

**Table 2:** List of protein functions identified from *Novosphingobium pentaromativorans* US6-1grown in the presence of PAHs.

Position	Protein Name	Accession No.	Species
Band 1	site-specific DNA-methyltransferase (adenine-specific)	gi|148556136|	*Sphingomonas wittichii* RW1
Band 1	3-oxoacyl-(acyl-carrier-protein) reductase	gi|56552118|	*Sphingomonas wittichii* RW1
Band 1	4-hydroxybenzoate 3-monooxygenase	gi|494072487|	*Novosphingobium pentaromativorans* US6-1
Band 1	homoserine kinase	gi|56552496|	*Sphingomonas wittichii* RW1
Band 2	acyl-CoA dehydrogenase-like protein	gi|103488374|	*Sphingopyxis alaskensis* RB2256
Band 2	thiol:disulfide interchange protein DsbC	gi|494068955|	*Novosphingobium pentaromativorans* US6-1
Band 2	salicylaldehyde dehydrogenase	gi|359402486|	*Novosphingobium pentaromativorans* US6-1
Band 2	SufBD	gi|87198224|	*Novosphingobium aromaticivorans* DSM 12444
Band 3	PAH ring-hydroxylating dioxygenase alpha subunit	gi|494073952|	*Novosphingobium pentaromativorans* US6-1
Band 3	4-oxalocrotonate decarboxylase	gi|148556423|	*Sphingomonas wittichii* RW1
Band 3	dihydrolipoamide dehydrogenase	gi|148550590|	*Sphingomonas wittichii* RW1
Band 3	hypothetical protein Swit_4997	gi|148550950|	*Sphingomonas wittichii* RW1
Band 3	hypothetical protein SKA58_01115	gi|94495050|	*Sphingomonas* sp. SKA58

The catalysis of 4-hydroxybenzoate into proto-catechuate by 4-hydroxybenzoate 3-monooxygenase involves the proto-catechuate branch of the 3-oxoadipate pathway. Following *ortho* cleavage of proto-catechuate by protocatechuate 3, 4-di-oxygenase and five additional enzymes convert the ring cleavage product to intermediates of the tricarboxylic acid (TCA) cycle. Many aromatic compounds under aerobic conditions are degraded by bacteria via the salicylate or proto-catechuate branch of the 3-oxoadipate pathway. The 3-oxoadipate pathway is biochemically conserved, and the structural genes encoding enzymes of this pathway are similar in widely differing bacterial species [26]–[27].

It is noteworthy that the pyridine-nucleotide-dependent reduction of a 3-oxoacyl-[acyl-carrier-protein] of the 3-oxoacyl-[acyl-carrier-protein] reductase, which catalyzes the first reductive step in *de novo* fatty acid biosynthesis, was identified in Band 1. This enzyme participates in fatty acid biosynthesis and polyunsaturated fatty acid biosynthesis. The composition of fatty acids can affect the fluidity of the cell membrane, and their metabolites also regulate the transcription of a variety of genes [28]. Furthermore, it has been suggested that 3-oxoacyl-[acyl-carrier-protein] reductase may play a role in the absorption of PAHs by changing the fluid of the cytomembrane. Homoserine kinase also was found in Band 1, which can catalyze ATP and L-homoserine to ADP and O-phospho-L-homoserine, and plays a role in glycine, serine, and threonine metabolism [29].

In Band 2, the detection of salicylaldehyde dehydrogenase may indicate the catalysis of salicylaldehyde into salicylate. This enzyme is involved in the catechol branch of the 3-oxoadipate pathway; therefore, *N. pentaromativorans* US6-1 may degrade PAH via the salicylate pathway by forming catechol, and then mineralizing this to CO_2_ via the TCA cycle [30]. In addition, three proteins whose functions have not been confirmed were found.

In Band 3, PAH RHDα, 4-oxalocrotonate decarboxylase, and dihydrolipoamide dehydrogenase were identified, and two unknown proteins were also found. The PAH RHDα is a component of the RHD enzyme system comprised of three components, in which an iron-sulfur flavoprotein reductase and an iron-sulfur ferredoxin transfer electrons from NAD(P)H to a terminal dioxygenase. The terminal dioxygenase is composed of large α and small β subunits. The alpha subunit (RHDα) contains two conserved regions: the [Fe2-S2] Rieske centre and the mononuclear iron-containing catalytic domain. Because of the hydrophobic nature and chemical resistance of PAHs, the initial step in their metabolism by RHDs is a difficult reaction, and is critical to the whole degradation process. In aerobic conditions, this initial step commonly occurs via the incorporation of molecular oxygen into the aromatic nucleus by a multicomponent RHD enzyme system, forming cis-dihydrodiol [31], [32]. The 4-oxalocrotonate decarboxylase, which decomposes the 4-oxalocrotonate to 2-oxopent-4-enoate and CO_2_, participates in the metabolic pathways of some benzenoids: benzoate, toluene, xylene and fluorene degradation [33].

Dihydrolipoamide dehydrogenase was also identified in Band 3, which was a catalytic component of the pyruvate dehydrogenase complex. It is a flavoprotein enzyme that degrades lipoamide, and produces dihydrolipoamide and acetyl-CoA, a key intermediate of carbon metabolism [34]. Another three proteins were detected in Band 3, but their functions were not confirmed.

The proteins involved in the self-protection, gene expression, metabolism of the carbon source and amino acid, and fatty acid biosynthesis that were differentially expressed between the cells growing on PAHs and the control indicate that growth on PAHs triggers a global change in cell physiology, and involves many cellular functions [35]. Since PAHs are found in planar, angular, or cluster arrangements, there are multiple sites of enzymatic attack, leading to diverse pathways and complex biochemical reactions that require diverse enzymes [36]. One example is the pyrene degradation pathway in *M. vanbaalenii* PYR-1 described by Kim et al [14]. In this pathway, degradation was initiated by the RHD to produce pyrene *cis*-1,2-dihydrodiol and pyrene *cis*-4,5-dihydrodiol, and the complete pyrene degradation pathway involved 27 enzymes and 25 steps. The proteins involved include 14 proteins responsible for the degradation of pyrene to phthalate, six proteins responsible for the degradation of phthalate to protocatechuate, seven proteins responsible for the lower pathway from protocatechuate to acetyl coenzyme A (acetylCoA) and succinyl-CoA, and three proteins responsible for the transformation of pyrene to 1,2-dimethoxypyrene. Madhumita [37] reported that the initial dioxygenation of phenanthrene occurred at both 3, 4- and 1, 2-carbon positions in *Sphingobium* sp. strain PNB. The further *meta*-cleavage of resultant diols led to the formation of 1-hydroxy-2-naphthoic acid and 2-hydroxy-1-naphthoic acid, respectively. These metabolites were subsequently transformed to catechol via salicylic acid, which is followed by 3-oxoadipate enol-lactone and then the TCA cycle, leading to complete mineralization. The 3-oxoadipate pathway is biochemically conserved, and the structural genes encoding enzymes of this pathway are similar among a variety of bacterial species [27]. Kim [38] reported that *N. pentaromativorans* US6-1 utilized two different extradiol pathways to degrade PAHs, one of which involves the enzyme salicylaldehyde dehydrogenase. Salicylaldehyde dehydrogenase was also identified in the Plasmid NSU_pLA1134 of *N. pentaromativorans* US6-1. Notably, PAH RHDα, 4-hydroxybenzoate 3-monooxygenase, and salicylaldehyde dehydrogenase were also found in the current study. This suggests that *N. pentaromativorans* US6-1 degraded PAHs via the metabolic route initiated by RHD; further degradation occurred via either *o*-phthalate pathway or salicylate pathway, and via *ortho-* or *meta-*cleavage to converge at the common intermediate 3-oxoadipate enol-lactone, resulting in TCA cycle intermediates, which finally were mineralized to CO_2_.

### 3 Relative gene expression following induction by three PAH compounds

The catabolic pathways for PAHs are complex, and differ among bacterial strains and under different environmental conditions. A substrate can occasionally be transformed into several intermediates under specific conditions. The recently isolated *Novosphingobium* is able to degrade a large spectrum of aromatic hydrocarbons, ranging from monocyclic to polycyclic hydrocarbons [39]–[41]. However, the genes for PAH degradation by *Novosphingobium* are little investigated and their characteristics are still obscure [42].

**Figure 3:**
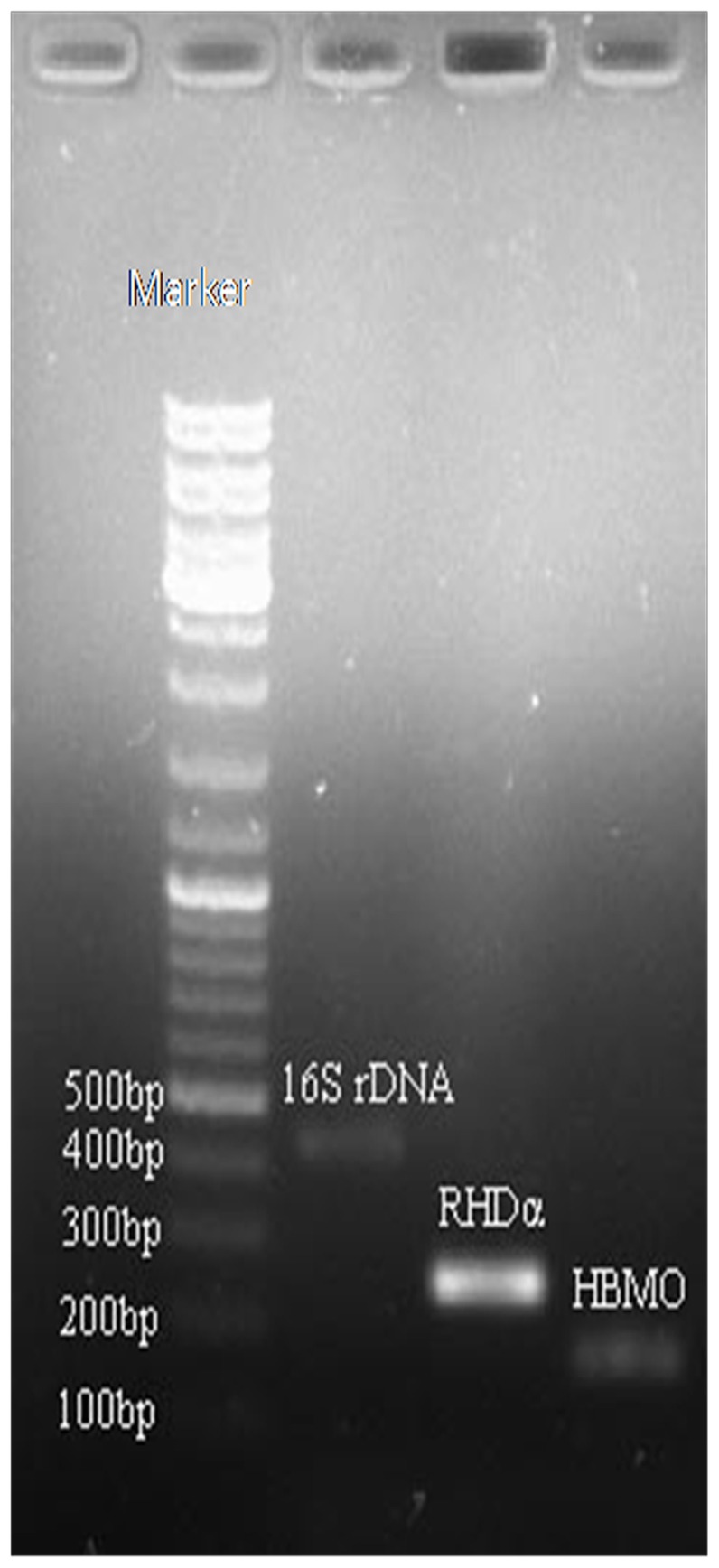
Real-Time PCR product of the 16S rRNA gene, PAH ring-hydroxylating dioxygenase, and 4-hydroxybenzoate 3-monooxygenase gene in *Novosphingobium pentaromativorans* US6-1.

In order to verify the results of protein identification and gain further insight to the mechanism of PAH mineralization in *N. pentaromativorans* US6-1, RHDα, and 4-hydroxybenzoate 3-monooxygenase, gene expression under induction by different PAHs in *N. pentaromativorans* US6-1 was investigated using Real-Time RT-PCR, and products were verified by gel electrophoresis. As shown in the Fig. 3, there were three bands coincident with the target fragments, 400–500 bp for 16S rRNA gene (433 bp), 200–300 bp for RHDα gene (252 bp), and 100–200 bp for 4-hydroxybenzoate 3-monooxygenase (HBMO) gene (157 bp).

**Figure 4:**
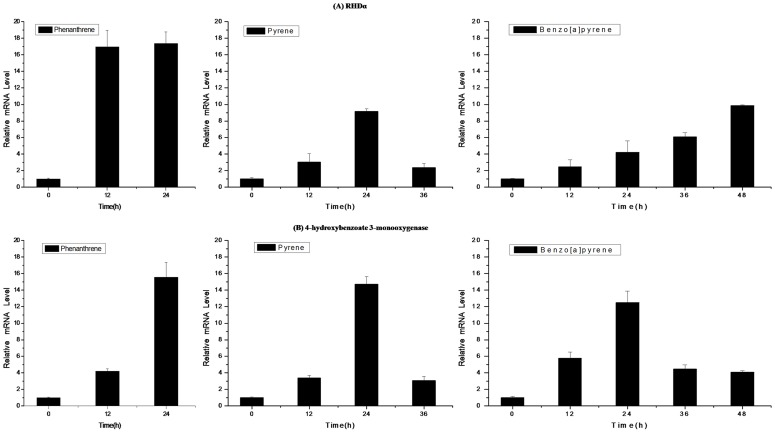
Relative quantification of RHDα (A) and 4-hydroxybenzoate 3-monooxygenase (B) gene expression in *Novosphingobium pentaromativorans* US6-1 following induction by three PAH compounds (mean and standard deviation of triplicate samples are shown).

As determined by real-time PCR analysis, levels of the RHDα gene in *N. pentaromativorans* US6-1 were variously upregulated in PAH-induced cultures compared with the control group. As shown in Fig. 4(A), we found that the gene expression of RHDα increased by approximately 17-fold compared with the control group 12 h or 24 h following induction by phenanthrene. This was consistent with the intensity of Band 3 in the SDS-PAGE pattern, and might be associated with the rapid degradation rate of phenanthrene. The gene expression of RHDα was upregulated and was highest (about 9-fold higher than the control) after 24 h, then decreased by 2.5-fold after induction by pyrene. This decline of RHDα after 24 h of pyrene induction may be related to inhibition by downstream reactions. However, when incubated in benzo[a]pyrene, its expression increased steadily to nearly 10-fold after 48 h of induction. This may be related to the slow and low degradation rate of benzo[a]pyrene, but indicated that the degradation of benzo[a]pyrene in US6-1 was initiated after 48 h incubation. Since benzo[a]pyrene is highly hydrophobic, mutagenic, and resistant to biodegradation, a longer time period is needed for degradation, and more genes and proteins are required [21], [36].

As shown in Fig. 4(B), the expression level of 4-hydroxybenzoate 3-monooxygenase was upregulated to different degrees after induction by phenanthrene, pyrene, and benzo[a]pyrene. Following exposure to phenanthrene, gene expression was 4.5-fold and 16-fold higher than the control at 12 h and 24 h, respectively. In the culture exposed to pyrene, this enzyme was upregulated to about 4-fold compared with the control. The highest level of upregulation observed was about 15-fold versus the control after 24 h induction, but this level subsequently decreased to 3.5-fold after 36 h exposure. When exposed to benzo[a]pyrene, expression of 4-hydroxybenzoate 3-monooxygenase was highest (approximately 13-fold) after 24 h. However, this level decreased to nearly 4-fold after 36 h and 48 h exposure. Interestingly, Band 2 was observed in the lanes of pyrene- and benzo[a]pyrene- induced cultures in which the salicylaldehyde dehydrogenase was found. This enzyme is involved in salicylate pathway, but was not detected during phenanthrene incubation. Unfortunately, we failed to amplify the gene of this enzyme in *N. pentaromativorans* US6-1; however, our results are otherwise comparable with data reported by Kim et al [38], which identified two different extradiol pathways that play a key role in the biodegradation of PAHs in *N. pentaromativorans* US6-1. Notably, salicylaldehyde dehydrogenase was previously identified in the plasmid NSU_pLA1134 of *N. pentaromativorans* US6-1.

The present study suggests that certain enzymes are shared in the mineralization pathways of PAHs. Some common enzymes in the catabolism of different PAHs have been reported, but it is reasonable to assume that exposure to various PAHs results in the initiation of different signals for the induction of proteins in *N. pentaromativorans* US6-1. Our hypothesis was based on the knowledge that *N. pentaromativorans* US6-1 has been exposed to a mixture of PAHs [15]. Over evolutionary time scales, such conditions would be expected to select for US6-1 with versatile metabolic capabilities. To conserve energy for vital growth-related processes, the enzymes needed to degrade a particular compound are not normally synthesized unless the compound is present in the medium. Therefore, the precise cellular response to a particular PAH could be specific to that form, thus enabling the identification of gene expression and the proteome characteristics associated with exposure.

## Conclusions

In conclusion, this investigation showed that *N. pentaromativorans* US6-1 is capable of degrading three- to five-ring PAHs, including phenanthrene, pyrene, and benzo[a]pyrene. When incubated with PAHs, multiple cellular functions and a global change in cell physiology were observed in response to PAH stress. *Novosphingobium pentaromativorans* US6-1 degraded PAHs via the metabolic route initiated by RHD, and further degradation occurred via either the *o*-phthalate pathway or the salicylate pathway [38]; both pathways subsequently entered the 3-oxoadipate pathway and TCA cycle, followed by mineralization to CO_2_. This investigation provides a basis for understanding the metabolic versatility of this bacterium in the degradation of other PAHs; however, cloning and characterization of catabolic genes and identification of key enzymes will enable the full characterization of PAHs metabolism of *N. pentaromativorans* US6-1.
