# Correction: Biodegradation of Polycyclic Aromatic Hydrocarbons by *Novosphingobium pentaromativorans* US6-1

**DOI:** 10.1371/journal.pone.0111319

**Published:** 2014-10-14

**Authors:** The PLOS ONE Staff

There is an error in Affiliation 1 for authors Yihua Lyu, Wei Zheng, Tianling Zheng, and Yun Tian. Affiliation 1 should be: Key Laboratory of the Ministry of Education for Coastal and Wetland Ecosystems, School of Life Science, Xiamen University, Xiamen, China.
